# Bis(2-amino-5-chloro­pyridinium) tetra­chloridozincate

**DOI:** 10.1107/S1600536811005691

**Published:** 2011-02-23

**Authors:** Riadh Kefi, Erwann Jeanneau, Frederic Lefebvre, Cherif Ben Nasr

**Affiliations:** a Laboratoire de Chimie des Matériaux, Faculté des sciences de Bizerte, 7021 Zarzouna, Tunisia; b Universtié Lyon1, Centre de Diffractométrie Henri Longchambon, 43 Boulevard du 11 Novembre 1918, 69622 Villeurbanne Cedex, France; c Laboratoire de Chimie Organometallique de Surface (LCOMS), Ecole Superieure de Chimie Physique Electronique, 69622 Villeurbanne Cedex, France

## Abstract

The asymmetric unit of the title compound, (C_5_H_6_ClN_2_)_2_[ZnCl_4_], contains two 2-amino-5-chloro­pyridinium cations and one [ZnCl_4_]^2−^ dianion which are held together by N—H⋯Cl and C—H⋯Cl hydrogen bonds. The [ZnCl_4_]^2−^ anions have a distorted tetra­hedral geometry. Weak inter­molecular π–π stacking inter­actions exist between neighbouring aromatic rings of the cations with a centroid–centroid distance of 3.712 (7) Å.

## Related literature

For common applications of organic–inorganic hybrid materials, see: Kobel & Hanack (1986 ▶); Pierpont & Jung (1994 ▶). For a related structure, see: Coomer *et al.* (2007 ▶). For π–π inter­actions between pyridinium cations, see: Albrecht *et al.* (2003 ▶). For aminium–iminium tautomerism, see: Jin *et al.* (2001 ▶). For a discussion of C—N—C pyridinium angles, see: Jin *et al.* (2005 ▶).
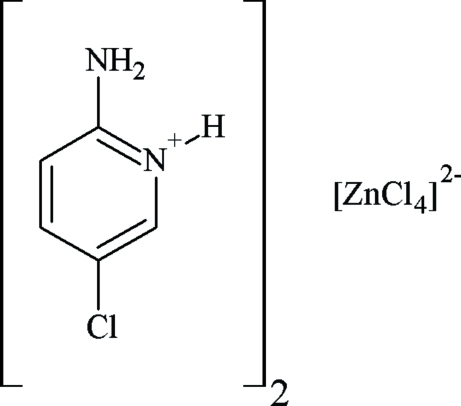


## Experimental

### 

#### Crystal data

(C_5_H_6_ClN_2_)_2_[ZnCl_4_]
*M* *_r_* = 466.33
Monoclinic, 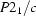

*a* = 13.317 (1) Å
*b* = 14.817 (2) Å
*c* = 8.571 (1) Å
β = 92.923 (9)°
*V* = 1689.0 (3) Å^3^
*Z* = 4
Mo *K*α radiation
μ = 2.40 mm^−1^
*T* = 110 K
0.23 × 0.15 × 0.10 mm

#### Data collection

Oxford Diffraction Xcalibur Atlas Gemini ultra diffractometer
Absorption correction: analytical *CrysAlis PRO* (Oxford Diffraction, 2009 ▶; Clark & Reid, 1995 ▶) *T*_min_ = 0.711, *T*_max_ = 0.835
22410 measured reflections
4360 independent reflections
3553 reflections with *I* > 2σ(*I*)
*R*_int_ = 0.061

#### Refinement

*R*[*F* ^2^ > 2σ(*F* ^2^)] = 0.051
*wR*(*F* ^2^) = 0.108
*S* = 1.02
4356 reflections
190 parameters
H-atom parameters constrained
Δρ_max_ = 1.27 e Å^−3^
Δρ_min_ = −1.03 e Å^−3^

### 

Data collection: *CrysAlis PRO* (Oxford Diffraction, 2009 ▶); cell refinement: *CrysAlis PRO*; data reduction: *CrysAlis PRO*; program(s) used to solve structure: *SIR97* (Altomare *et al.*, 1999 ▶); program(s) used to refine structure: *CRYSTALS* (Betteridge *et al.*, 2003 ▶); molecular graphics: *CAMERON* (Watkin *et al.*, 1996 ▶); software used to prepare material for publication: *CRYSTALS*.

## Supplementary Material

Crystal structure: contains datablocks global, I. DOI: 10.1107/S1600536811005691/cv5049sup1.cif

Structure factors: contains datablocks I. DOI: 10.1107/S1600536811005691/cv5049Isup2.hkl

Additional supplementary materials:  crystallographic information; 3D view; checkCIF report

## Figures and Tables

**Table 1:** Hydrogen-bond geometry (Å, °)

*D*—H⋯*A*	*D*—H	H⋯*A*	*D*⋯*A*	*D*—H⋯*A*
N19—H191⋯Cl2^i^	0.85	2.76	3.496 (5)	146
N20—H201⋯Cl2^i^	0.85	2.41	3.231 (6)	163
N19—H192⋯Cl3^ii^	0.85	2.75	3.491 (5)	146
C17—H171⋯Cl3^ii^	0.92	2.65	3.442 (8)	144
N9—H91⋯Cl4^iii^	0.86	2.38	3.197 (4)	157
N11—H112⋯Cl4^iii^	0.87	2.58	3.356 (4)	148
N11—H111⋯Cl5^iv^	0.86	2.45	3.291 (6)	165

Symmetry codes: (i) 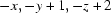; (ii) 
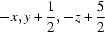
; (iii) 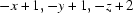; (iv) 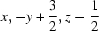.

**Fig. 1.**
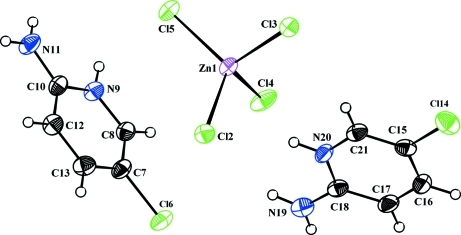
View of (I), showing 50% probability displacement ellipsoids and arbitrary spheres for the H atoms. Dashed lines denote hydrogen bonds.

## supplementary crystallographic information

### Comment

Organic-inorganic hybrid materials have been extensively studied in recent years due to their potential applications in various field (Kobel & Hanack, 1986; Pierpont & Jung, 1994). Herewith we report the crystal structure of the title compound, (I), formed in the reaction of 2-amino-5-chloropyridine with zinc chloride. The crystal structure of hydrated form of (I)  (CCDC refcode JIPHAS) was reported recently by Coomer *et al.* (2007) .

In (I) (Fig.1), only the nitrogen atom of the aromatic ring of the title compound is protonated but not the amino group. Thus, to ensure charge equilibrium, the structure associates one tetrachlorozincate dianion with two 2-amino-5-chloropyridinium cations. The atomic arrangement of the title hybrid material can be described as inorganic [ZnCl_4_]^2-^ units separated by the organic cations. The different entities are held together by columbic attraction and multiple hydrogen bonds to form a three dimensional network.  The organic cations and inorganic dianion sare form N—H···Cl and C—H···Cl hydrogen bonds (Table 1). Intermolecular π-π interaction is present between identical antiparallel 2-amino-5-chloropyridinium cations with the centroid-to-centrpoid separation of 3.712 (7) Å. This π-stacking between pyridinium cations is weaker than that in bis(2-amino-5-methylpyridinium) tetrachlorozincate where the longest distance between the centroids is 3.54 Å (Albrecht *et al.*, 2003). In the organic entity, the N11—C10 bond [1.332 (6) Å] is shorter than the N9—C10 [1.347 (6) Å] and N9—C8 [1.357 (6) Å] bonds, consistent with the iminuim tautomer (Jin *et al.*, 2001). Moreover, the existence of the iminuim tautomer is supported by the fact that the C10—C12 [1.408 (6) Å] and C7—C13 [1.408 (7) Å] bonds are longer than the C12—C13 [1.372 (7) Å] and C7—C8 [1.344 (6) Å] bonds. Similar features are also observed in the other organic cations. However, previous study show that a pyridinium cation always possesses an expanded angle of C—N—C in comparison with the parent pyridine (Jin *et al.*, 2005).

### Experimental

A mixture of aqueous solution of 2-amino-5-chloropyridine (3 mmol, 0.385 g), zinc chloride (1.5 mmol, 0.297 g) and HCl (10 ml, 0.3 *M*) in a Petri dish was slowly evaporated at room temperature. Colourless single crystals of the title compound were isolated after several days (yield 54%).

### Refinement

All H atoms were initially located in a difference map, but placed in idealized positions (C—H  0.93–0.98 Å, N—H  0.86–0.89 Å) and refined as riding, with *U*_iso_(H) = 1.2–1.5 *U*_eq_ of the parent atom.

### Crystal data

**Table:**

(C_5_H_6_ClN_2_)_2_[ZnCl_4_]	*F*(000) = 928
*M**_r_* = 466.33	*D*_x_ = 1.834 Mg m^−^^3^
Monoclinic, *P*2_1_/*c*	Mo *K*α radiation, λ = 0.7107 Å
Hall symbol: -P 2ybc	Cell parameters from 8999 reflections
*a* = 13.317 (1) Å	θ = 3.4–29.5°
*b* = 14.817 (2) Å	µ = 2.40 mm^−^^1^
*c* = 8.571 (1) Å	*T* = 110 K
β = 92.923 (9)°	Block, colourless
*V* = 1689.0 (3) Å^3^	0.23 × 0.15 × 0.10 mm
*Z* = 4	

### Data collection

**Table:**

Oxford Diffraction Xcalibur Atlas Gemini ultra diffractometer	4360 independent reflections
Radiation source: Enhance (Mo) X-ray Source	3553 reflections with *I* > 2σ(*I*)
graphite	*R*_int_ = 0.061
Detector resolution: 10.4685 pixels mm^-1^	θ_max_ = 29.6°, θ_min_ = 3.4°
ω scans	*h* = −18→18
Absorption correction: analytical *CrysAlis PRO* (Oxford Diffraction, 2009; Clark & Reid, 1995)	*k* = −20→18
*T*_min_ = 0.711, *T*_max_ = 0.835	*l* = −11→11
22410 measured reflections	

### Refinement

**Table:**

Refinement on *F*^2^	Primary atom site location: structure-invariant direct methods
Least-squares matrix: full	Hydrogen site location: inferred from neighbouring sites
*R*[*F*^2^ > 2σ(*F*^2^)] = 0.051	H-atom parameters constrained
*wR*(*F*^2^) = 0.108	Method, part 1, Chebychev polynomial [Watkin, D. J. (1994). *Acta Cryst.* A50, 411–437; *P*rince, E. (1982). Mathematical Techniques in Crystallography and Materials Science Springer-Verlag, Ne*w* York.] [*w*eight] = 1.0/[A_0_*T_0_(x) + A_1_*T_1_(x) ··· + A_n-1_]*T_n-1_(x)] where A_i_ are the Chebychev coefficients listed belo*w* and x = *F* /*F*max Method = Robust Weighting (*P*rince, 1982) W = [*w*eight] * [1-(delta*F*/6*sigma*F*)^2^]^2^ A_i_ are: 425. 613. 311. 83.3
*S* = 1.02	(Δ/σ)_max_ = 0.001
4356 reflections	Δρ_max_ = 1.27 e Å^−^^3^
190 parameters	Δρ_min_ = −1.03 e Å^−^^3^
0 restraints	

### Fractional atomic coordinates and isotropic or equivalent isotropic displacement parameters (Å^2^)

**Table:**

	*x*	*y*	*z*	*U*_iso_*/*U*_eq_	
Zn1	0.25192 (4)	0.48608 (3)	1.01057 (6)	0.0183	
Cl2	0.15870 (8)	0.47893 (8)	0.78203 (13)	0.0220	
Cl3	0.13940 (9)	0.48449 (8)	1.19926 (13)	0.0255	
Cl4	0.34397 (10)	0.35524 (8)	1.02302 (17)	0.0298	
Cl5	0.35902 (9)	0.60259 (8)	1.04980 (15)	0.0249	
Cl6	0.33629 (9)	0.37113 (8)	0.53253 (15)	0.0266	
C7	0.3767 (3)	0.4786 (3)	0.5834 (6)	0.0213	
C8	0.4484 (3)	0.4888 (3)	0.6981 (5)	0.0204	
N9	0.4826 (3)	0.5725 (3)	0.7372 (5)	0.0201	
C10	0.4457 (3)	0.6486 (3)	0.6703 (6)	0.0194	
N11	0.4816 (3)	0.7281 (3)	0.7197 (5)	0.0263	
C12	0.3706 (3)	0.6396 (3)	0.5495 (6)	0.0218	
C13	0.3361 (4)	0.5555 (3)	0.5071 (6)	0.0242	
H131	0.2865	0.5492	0.4259	0.0299*	
H121	0.3449	0.6907	0.4991	0.0261*	
H91	0.5254	0.5774	0.8160	0.0238*	
H81	0.4748	0.4393	0.7511	0.0250*	
Cl14	0.18546 (9)	0.74583 (10)	0.78663 (15)	0.0313	
C15	0.0852 (3)	0.7508 (3)	0.9057 (5)	0.0221	
C16	0.0460 (4)	0.8350 (3)	0.9491 (6)	0.0253	
C17	−0.0331 (4)	0.8381 (3)	1.0446 (6)	0.0247	
C18	−0.0742 (3)	0.7580 (3)	1.1000 (5)	0.0201	
N19	−0.1504 (3)	0.7566 (3)	1.1978 (5)	0.0249	
N20	−0.0342 (3)	0.6791 (3)	1.0543 (5)	0.0211	
C21	0.0433 (4)	0.6741 (3)	0.9572 (5)	0.0211	
H211	0.0658	0.6181	0.9267	0.0262*	
H201	−0.0611	0.6300	1.0824	0.0252*	
H171	−0.0594	0.8932	1.0719	0.0302*	
H161	0.0734	0.8879	0.9135	0.0311*	
H191	−0.1768	0.7063	1.2220	0.0299*	
H192	−0.1759	0.8065	1.2265	0.0297*	
H111	0.4608	0.7769	0.6733	0.0310*	
H112	0.5310	0.7303	0.7899	0.0310*	

### Atomic displacement parameters (Å^2^)

**Table:**

	*U*^11^	*U*^22^	*U*^33^	*U*^12^	*U*^13^	*U*^23^
Zn1	0.0174 (2)	0.0137 (2)	0.0236 (3)	−0.00055 (19)	−0.00135 (19)	−0.0007 (2)
Cl2	0.0220 (5)	0.0207 (5)	0.0231 (5)	0.0011 (4)	−0.0009 (4)	−0.0009 (4)
Cl3	0.0301 (6)	0.0239 (5)	0.0229 (5)	−0.0059 (5)	0.0040 (4)	−0.0019 (4)
Cl4	0.0291 (6)	0.0153 (5)	0.0433 (7)	0.0046 (4)	−0.0137 (5)	−0.0039 (5)
Cl5	0.0241 (5)	0.0182 (5)	0.0322 (6)	−0.0055 (4)	−0.0004 (4)	−0.0025 (4)
Cl6	0.0257 (6)	0.0180 (5)	0.0363 (6)	−0.0038 (4)	0.0033 (5)	−0.0068 (5)
C7	0.021 (2)	0.0142 (19)	0.030 (2)	0.0002 (17)	0.0046 (18)	−0.0035 (18)
C8	0.026 (2)	0.0135 (19)	0.022 (2)	0.0044 (17)	−0.0003 (17)	0.0009 (16)
N9	0.0188 (18)	0.0164 (18)	0.025 (2)	0.0020 (14)	−0.0030 (15)	−0.0001 (15)
C10	0.019 (2)	0.0136 (19)	0.026 (2)	0.0032 (16)	0.0013 (17)	0.0001 (17)
N11	0.032 (2)	0.0140 (18)	0.032 (2)	0.0001 (16)	−0.0068 (18)	0.0007 (16)
C12	0.021 (2)	0.018 (2)	0.026 (2)	0.0048 (17)	−0.0008 (18)	0.0017 (18)
C13	0.023 (2)	0.023 (2)	0.025 (2)	0.0020 (18)	−0.0043 (18)	−0.0022 (19)
Cl14	0.0248 (6)	0.0439 (7)	0.0252 (6)	−0.0010 (5)	0.0001 (4)	−0.0005 (5)
C15	0.022 (2)	0.026 (2)	0.018 (2)	−0.0015 (18)	−0.0036 (17)	−0.0035 (18)
C16	0.033 (3)	0.019 (2)	0.024 (2)	−0.0047 (19)	−0.001 (2)	0.0014 (18)
C17	0.037 (3)	0.013 (2)	0.024 (2)	0.0019 (18)	−0.003 (2)	−0.0020 (17)
C18	0.019 (2)	0.019 (2)	0.022 (2)	0.0006 (17)	−0.0038 (17)	−0.0028 (17)
N19	0.025 (2)	0.023 (2)	0.027 (2)	0.0019 (16)	−0.0022 (16)	−0.0040 (17)
N20	0.024 (2)	0.0138 (17)	0.025 (2)	−0.0028 (15)	0.0017 (16)	−0.0010 (15)
C21	0.025 (2)	0.0147 (19)	0.023 (2)	0.0030 (17)	−0.0068 (18)	−0.0013 (17)

### Geometric parameters (Å, °)

**Table:**

Zn1—Cl2	2.2674 (12)	C12—H121	0.928
Zn1—Cl3	2.2602 (13)	C13—H131	0.939
Zn1—Cl4	2.2934 (13)	Cl14—C15	1.723 (5)
Zn1—Cl5	2.2535 (12)	C15—C16	1.410 (7)
Cl6—C7	1.730 (5)	C15—C21	1.351 (7)
C7—C8	1.344 (6)	C16—C17	1.367 (7)
C7—C13	1.408 (7)	C16—H161	0.923
C8—N9	1.357 (6)	C17—C18	1.400 (7)
C8—H81	0.922	C17—H171	0.923
N9—C10	1.347 (6)	C18—N19	1.349 (6)
N9—H91	0.864	C18—N20	1.351 (6)
C10—N11	1.332 (6)	N19—H191	0.854
C10—C12	1.408 (6)	N19—H192	0.855
N11—H111	0.863	N20—C21	1.361 (6)
N11—H112	0.869	N20—H201	0.852
C12—C13	1.372 (7)	C21—H211	0.924
			
Cl2—Zn1—Cl3	105.30 (5)	C7—C13—C12	119.8 (4)
Cl2—Zn1—Cl4	105.57 (5)	C7—C13—H131	120.1
Cl3—Zn1—Cl4	109.28 (5)	C12—C13—H131	120.1
Cl2—Zn1—Cl5	118.63 (5)	Cl14—C15—C16	120.2 (4)
Cl3—Zn1—Cl5	109.81 (5)	Cl14—C15—C21	120.2 (4)
Cl4—Zn1—Cl5	107.93 (5)	C16—C15—C21	119.5 (4)
Cl6—C7—C8	119.2 (4)	C15—C16—C17	119.7 (4)
Cl6—C7—C13	121.4 (4)	C15—C16—H161	120.4
C8—C7—C13	119.4 (4)	C17—C16—H161	119.9
C7—C8—N9	120.0 (4)	C16—C17—C18	120.1 (4)
C7—C8—H81	120.7	C16—C17—H171	119.7
N9—C8—H81	119.3	C18—C17—H171	120.3
C8—N9—C10	123.4 (4)	C17—C18—N19	122.9 (4)
C8—N9—H91	118.0	C17—C18—N20	117.9 (4)
C10—N9—H91	118.3	N19—C18—N20	119.1 (4)
N9—C10—N11	119.2 (4)	C18—N19—H191	119.7
N9—C10—C12	117.6 (4)	C18—N19—H192	119.2
N11—C10—C12	123.1 (4)	H191—N19—H192	120.7
C10—N11—H111	119.5	C18—N20—C21	123.2 (4)
C10—N11—H112	120.0	C18—N20—H201	118.8
H111—N11—H112	120.1	C21—N20—H201	117.9
C10—C12—C13	119.8 (4)	N20—C21—C15	119.6 (4)
C10—C12—H121	119.8	N20—C21—H211	119.3
C13—C12—H121	120.4	C15—C21—H211	121.1

### Hydrogen-bond geometry (Å, °)

**Table:**

*D*—H···*A*	*D*—H	H···*A*	*D*···*A*	*D*—H···*A*
N19—H191···Cl2^i^	0.85	2.76	3.496 (5)	146
N20—H201···Cl2^i^	0.85	2.41	3.231 (6)	163
C21—H211···Cl2	0.92	2.73	3.635 (6)	165
N19—H192···Cl3^ii^	0.85	2.75	3.491 (5)	146
C13—H131···Cl3^iii^	0.94	2.85	3.772 (5)	166
C16—H161···Cl3^iv^	0.92	2.81	3.682 (7)	158
C17—H171···Cl3^ii^	0.92	2.65	3.442 (8)	144
N9—H91···Cl4^v^	0.86	2.38	3.197 (4)	157
N11—H112···Cl4^v^	0.87	2.58	3.356 (4)	148
N11—H111···Cl5^iv^	0.86	2.45	3.291 (6)	165
C8—H81···Cl5^v^	0.92	2.79	3.538 (4)	138

Symmetry codes: (i) −*x*, −*y*+1, −*z*+2; (ii) −*x*, *y*+1/2, −*z*+5/2; (iii) *x*, *y*, *z*−1; (iv) *x*, −*y*+3/2, *z*−1/2; (v) −*x*+1, −*y*+1, −*z*+2.
